# False failures, real distrust: the impact of an infrastructure failure deepfake on government trust

**DOI:** 10.3389/fpsyg.2025.1574840

**Published:** 2025-05-23

**Authors:** Saifuddin Ahmed, Muhammad Masood, Adeline Wei Ting Bee, Kei Ichikawa

**Affiliations:** ^1^Wee Kim Wee School for Communication and Information, Nanyang Technological University, Singapore, Singapore; ^2^Computational Social Science, Tokyo Institute of Technology, Tokyo, Japan

**Keywords:** deepfakes, cognitive ability, experiment, disinformation, political trust, government, deep fake, misinformation

## Abstract

Deepfakes today represent a novel threat that can induce widespread distrust more effectively than traditional disinformation due to its potential for greater susceptibility. In this study, we specifically test how individuals' exposure to deepfakes related to public infrastructure failures is linked to distrust in government, with their cognitive reflection and education possibly acting as a buffer. Using experimental data from the United States and Singapore, our findings indicate that exposure to deepfakes depicting a localized infrastructure failure, i.e., the collapse of a public bridge, heightens distrust in government among American participants but not Singaporeans. Additionally, education was found to be a significant moderator such that higher education levels is associated with lower political distrust when exposed to deepfakes. The role of deepfakes in influencing distrust in the government and the broader implications of these findings are discussed.

## 1 Introduction

The proliferation of online disinformation, particularly deepfakes, poses significant threats to the fabric of different societies worldwide (Westerlund, 2019). Research shows that disinformation erodes individuals' sense of reality and trust (Lewandowsky et al., 2017). With generative AI (GenAI) enabling faster, cheaper, more convincing, and tailored disinformation, scholars and policymakers are urgently seeking ways to coordinate efforts to regulate and mitigate the impact of deepfakes. Previous studies have identified key cognitive, media use, and demographic factors that shape attitudes and behaviors toward disinformation, including deepfakes (Ahmed and Chua, 2023; Nas and De Kleijn, 2024). For example, individuals with high emotionality and intuitive cognitive styles—compared to those with more rational styles—are more susceptible to disinformation (e.g., Bago et al., 2020; Pennycook and Rand, 2019a). Moreover, active social media users are more likely to trust and share disinformation than inactive users (Ahmed and Rasul, 2022), this may increase the risk of deepfake engagement as well. Age also emerges as a significant demographic risk factor, with older individuals showing higher vulnerability to deepfakes (Doss et al., 2023).

In this study, we investigate the civic impact of highly contextualized deepfakes across two politically and culturally distinct settings: the U.S. and Singapore. The widespread accessibility of deepfake production tools and the rapid dissemination of deceptive content motivated us to examine the causal effects of context-specific deepfakes (e.g., public infrastructure failures) on distrust in government using an experimental design. Specifically, the political and media contexts shape citizens' susceptibility to misinformation and political distrust (Flynn et al., 2017; Lazer et al., 2018). In the U.S., a fragmented and polarized media ecosystem amplifies partisan narratives and primes citizens to be more sensitive to perceived failures in governance, particularly in domains like infrastructure that are politically salient and tied to tax spending (Prior, 2013; Tsfati and Cappella, 2003). This aligns with the broader literature that citizens in more polarized or adversarial information environments are more susceptible to political distrust when exposed to disinformation (Flynn et al., 2017; Lazer et al., 2018). In contrast, Singapore's media system is more centralized and state-aligned, fostering high institutional trust and consistent narratives of government competence (Edelman, 2024; Pandian and McGonigle, 2024). These differences provide an important context for understanding how deepfake exposure may differentially influence trust in government across national settings. Thus, these two countries were selected to capture cross-national variation in media systems and political cultures.

Moreover, infrastructure failure serves as a highly visible and symbolic indicator of government competence, making it a compelling domain for examining how deepfakes may influence institutional trust. Perceptions of government responsibility are often shaped by performance in service delivery areas such as transportation, where citizens can directly observe outcomes (Hetherington, 2005; Rudolph and Evans, 2005). A fabricated failure in this domain—such as a collapsed bridge—can plausibly activate blame attributions and negative evaluations of state effectiveness. By leveraging a localized deepfake of infrastructure collapse, this study probes how disinformation embedded in salient policy domains can erode public trust in government, particularly in contexts where performance expectations are politically charged.

Therefore, we rely on responsibility attribution theory to rationalize the underpinning link between exposure to deepfakes of public infrastructure failures and mistrust specifically toward the government (Weiner, 2012). It suggests that individuals evaluate political actors based on perceived responsibility for outcomes, especially in visible domains like public service or crisis management. Additionally, we explore whether cognitive ability—identified in a number of prior research as a key protective factor against disinformation (e.g., Ahmed, 2021a,b; Brashier and Schacter, 2020)—can mitigate the expected negative effects of deepfakes on trust in government. It refers to an individual's general capacity for reasoning, problem-solving, and processing information—often linked to analytical thinking and reflective judgment (Stanovich and West, 2000). The following sections provide an overview of the importance of trust in society and focus on the specific role of deepfake exposure in eroding trust in government. We then discuss cognitive ability as a potential buffer against the negative effect of deepfakes, followed by a detailed discussion of our methodology, results, and conclusions.

### 1.1 Trust in government and the role of deepfakes

Trust in government—defined as the confidence of citizens as well as businesses in the policies of government to do what is right and fair—is foundational for any well-functioning society (Cook, 2001). The American National Election Study operationalizes it as how much individuals trust the government to “do the right thing” (Gershtenson and Plane, 2007). Without trust, countries and jurisdictions face significant pushback even when meting out sound policies. When collaborations are large-scale, trust becomes more vital, and authorities like government officials, state representatives, or politicians determine the success of adherence to social norms (Harring et al., 2021; Norenzayan and Shariff, 2008). For example, governmental trust was found to influence the attitude and uptake of COVID-19 prevention behaviors like social distancing, mask-wearing, and vaccine adoption (Liu et al., 2021; Vardavas et al., 2021).

Thus, with lack of trust in government, the public will put excessive burdens on policymakers to justify decisions and be slow or resistant to comply. Not only does this create an impasse, but it also increases the cost of political governance (Fan et al., 2022). Recent times have made (mis)trust in government particularly salient. Crises, such as the COVID-19 pandemic, the Russo-Ukrainian war, and the Israel-Hamas conflict, exemplify the need for trust to achieve swift, efficient, and coordinated national and international responses. It is evident that deep fissures in trust between citizens and their government exist across numerous societies and in crises, leaders are often faced with the challenge of rallying quick consensus amidst highly polarized opinions, widespread disinformation, and deep suspicion against institutions and authorities (Roozenbeek et al., 2020).

In this regard, a new tool malicious actors are using to undermine trust in government is the dessimination of localized and coustomized political deepfakes to threaten governments' legitimacy. Political deepfakes refer to synthetically generated audiovisual media with political connotations of events or people doing or saying things that never happened (Godulla et al., 2021). Often deepfakes are found to polarize and deepen misunderstanding among citizens (Walker, 2019; Westerlund, 2019). Unlike simple textual disinformation, deepfakes may be persuasive because they are hyper-realistic “evidence” that are not easily distinguishable from reality.

We aim to focus on a relatively understudied subset of deepfakes, which is more insidious and likely to undermines trust—that is deepfakes attacking the government's competencies in a seemingly objective way by fabricating public good failures or poor governance in different public sectors. Compared to deepfakes targeting politicians and personalities, these deepfakes do not appear incendiary on the surface but plant doubt on the reliability and competence of the government. The prerequisites to trust often hinge upon two key factors, a history of reliable public goods and services and a fair distribution of them among various social classes (Rothstein and Uslaner, 2005). Thus, when exposed to deepfakes of infrastructural failures, that is, public goods and services, individuals may show distrust in their government. Previous research has shown that government scandals and poor economic performance contribute to declining public trust in government (Chanley et al., 2000).

Moreover, drawing on responsibility attribution theory (Weiner, 2012), we argue that the public often perceives public safety as a core responsibility of the government. Thus, in cases of serious crises or failures, such as public infrastructure collapse or poor quality, the government is often held accountable for oversight, regulation, and response efforts (Boin et al., 2008). This tendency can explain why mistrust in government becomes a focal construct in public perception, rather than trust in private entities like companies. Furthermore, this is amplified by the symbolic role governments play in safeguarding societal welfare, which positions them as the ultimate custodians of accountability (Hood, 2011). Failures in oversight are often perceived as systemic issues rather than isolated incidents, deepening public skepticism toward governmental institutions (Bovens, 2007). Media framing also exacerbates this perception by highlighting governmental responsibility, especially in high-stakes crises (Iyengar, 1994; Entman, 1993). Additionally, the public's perception of government accountability is influenced by institutional trust, which is shaped by historical experiences of governance and crisis management efficacy (Rothstein and Stolle, 2008). Individuals also elaborate on availability heuristics, relying mental shortcuts (Tversky and Kahneman, 1973), which shape their opinion by signaling the government as the primarily responsible actor, particularly when such narratives dominate public discourse like media coverage. Based on the above discussion, we propose the following hypothesis:

**H1**: Exposure to deepfakes of infrastructural failures increases citizens' mistrust of the government.

### 1.2 Potential role of cognitive ability

Next, we investigate how cognitive ability, a general intelligence trait, fares against the effects of deepfakes. Several studies have shown how cognitive ability mitigates deepfake susceptibility (Ahmed, 2021a,b; Ask et al., 2023), yet others have reported weak or null effects (Lewandowsky et al., 2017). It suggests that the construct has theoretical relevance and empirical precedence in the literature on disinformation (De Keersmaecker and Roets, 2017; Pennycook and Rand, 2019a). Other potential moderators, such as political ideology, were not considered because they lacked sufficient theoretical backing for this study's specific comparative focus or in other words, did not align directly with the cross-societal nature of our study.

Cognitive ability may play a crucial role in mitigating the effects of deepfakes on trust in government for several reasons. First, individuals with higher cognitive skills are generally better equipped to critically evaluate information and discern credible sources from misleading ones (Pennycook and Rand, 2019b). This critical thinking enables them to approach media, including deepfake content, with skepticism, prompting them to verify the authenticity of content before forming opinions or sharing it further. For example, research indicates that cognitive complexity is positively correlated with the ability to navigate complex information environments, which is essential in an era rife with manipulated media (Pennycook and Rand, 2019a). Cognitive complexity, in contrast, refers to the degree to which individuals perceive and interpret the world in nuanced and differentiated ways (Conway et al., 2008). While related, it emphasizes open-mindedness and integrative thinking more than raw analytic capacity.

Second, individuals with strong cognitive abilities tend to possess greater media literacy, which includes understanding the techniques behind digital manipulation and the potential for disinformation (Ahmed and Chua, 2023; Brashier and Schacter, 2020). This awareness empowers them to recognize the hallmarks of deepfake content, such as inconsistencies in visual or auditory cues, thereby reducing the likelihood of being deceived (Ahmed and Chua, 2023; Brashier and Schacter, 2020).

Third, individuals with higher cognitive functioning are more likely to engage in reflective thinking and seek out multiple sources of information to form well-rounded perspectives (Stanovich and West, 2008). This tendency reduces reliance on single pieces of media, thereby diminishing the impact of any one deepfake on their trust in government. By evaluating information from various viewpoints and considering the broader context of a situation, cognitively adept individuals may maintain a more stable trust in government institutions, even in the face of potential manipulations. This multi-faceted engagement with information is likely to encourage a more nuanced understanding of political narratives and reduce susceptibility to sensationalized or distorted representations of reality. Collectively, these factors suggest that cognitive ability offers protection against the corrosive effects of deepfake technology on civic trust, fostering a more discerning citizenry capable of navigating the complexities of contemporary media landscapes.

However, some research reveals that this is not always the case. First, even individuals with high cognitive abilities can experience cognitive overload when confronted with an overwhelming amount of complex information (Sweller et al., 2011). The rapid proliferation of deepfake content, combined with the intricate nature of media manipulation, can easily overwhelm critical thinking processes. As a result, even the most cognitively adept individuals may struggle to consistently analyze and verify the authenticity of the information, increasing their susceptibility (Lewandowsky et al., 2017). Second, regardless of their cognitive skills, individuals often exhibit confirmation bias, which leads them to favor information that aligns with their preexisting beliefs (Pennycook and Rand, 2019a). This bias can prevent even the most critical thinkers from objectively evaluating deepfake content, especially if it resonates with their political or social viewpoints. Consequently, deepfakes that align with one's beliefs may be accepted without scrutiny, undermining the potential mitigating effects of cognitive ability (Pennycook and Rand, 2019a).

Therefore, given a contrary argument regarding the role of cognitive ability in the disinformation literature, we propose the following research question instead of a hypothesis:

***RQ1***: How does cognitive ability moderate the effect of exposure to deepfakes of infrastructural failures on citizens' distrust of the government?

## 2 Materials and method

### 2.1 Sample

The study enlisted an online panel platform—Qualtrics LLC—to recruit participants from the US (*N* = 303) and Singapore (*N* = 310) in June 2023. The samples were obtained using a quota sampling method, which matched the samples by age and gender. This was done to ensure the generalizability of the findings to the respective national populations. The sample also meets the criteria for power analyses (see Supplementary material A). The study was approved by the institutional review board at host university. All participants provided informed consent prior to participation.

### 2.2 Procedure

We employed a experiment framework in which participants were led to unique survey links. Participants were randomly assigned to either the experimental or control conditions within each country sample. In the experimental condition, participants were exposed to a deepfake of a collapsed bridge. As such, we included an experimental design consisting of a control (or baseline) condition (no deepfakes were shown) and an experimental condition (deepfake published on social media).

These deepfake images were created and customized to the respective country contexts. The specific location of the bridge collapse was decided based on a few considerations, including a relatively broad and vague region, e.g., “west of Singapore” and “Detroit, the US.” This was done to avoid using a single stimulus across countries and simultaneously test the power of contextualized deepfakes.

### 2.3 Measures

In the first step of the experiment, participants answered standard demographic questions, including age (the US, *M* = 48.50, *SD* = 15.91; Singapore, *M* = 41.64, *SD* = 13.70), gender (the US, female 53%; Singapore female 47%), education (the US, *M* = 4.97, *SD* = 1.58; Singapore, *M* = 5.42, *SD* = 1.17), and income (the US, *M* = 6.32, *SD* = 3.98; Singapore, *M* = 5.56, *SD* = 2.62).

**Figure 1:**
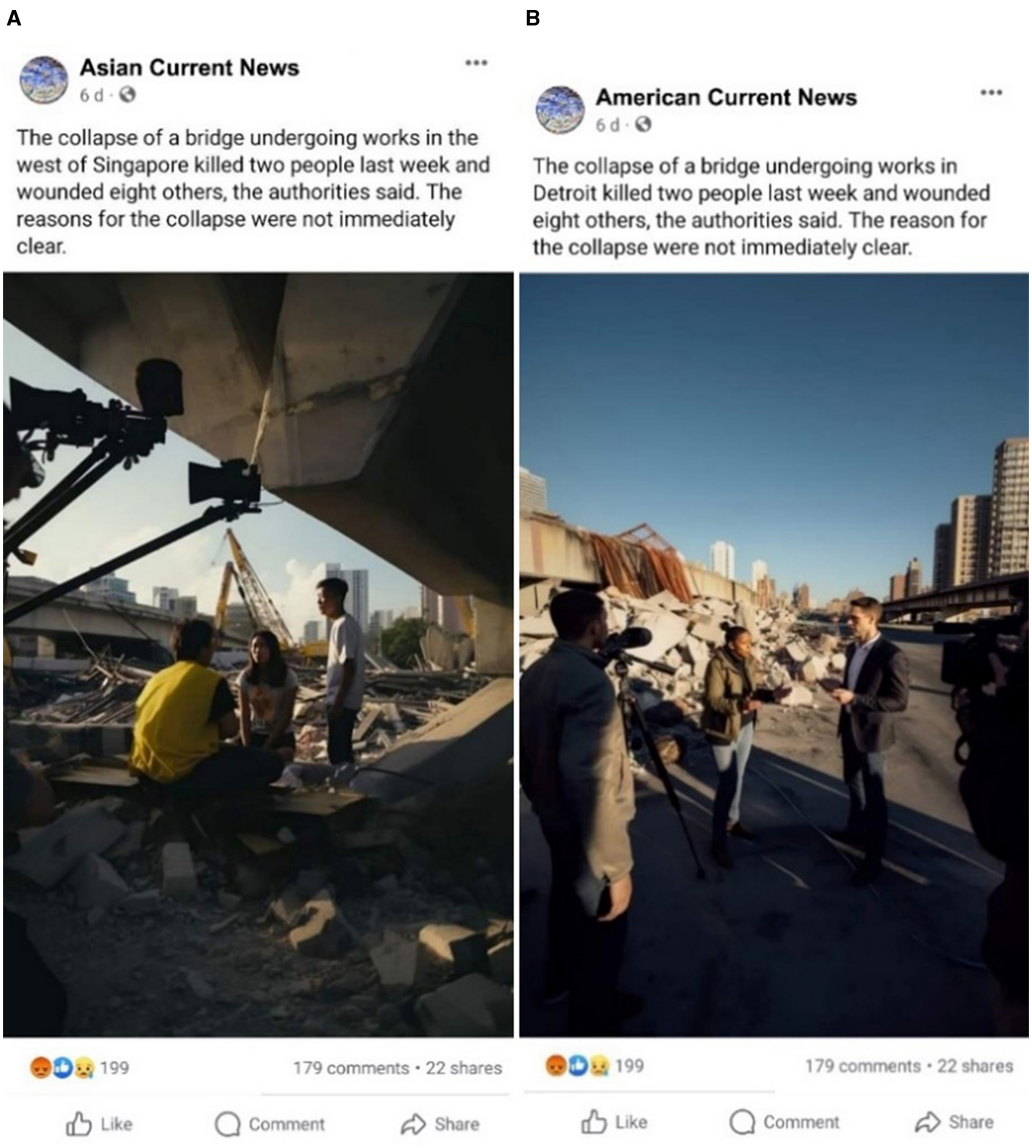
Deepfake images for Singapore **(A)** and the US **(B)**. These images are AI generated.

The second step was exposing participants in the treatment group to deepfakes of bridge collapse. The following are the two stimuli used in this study, and a caption describes the context of each stimulus (Figures 1A, B). Participants in the control group were not exposed to any stimulus.

In the final step, participants in both groups responded to questions about their trust in the government. Participants were asked to rate the statement, “I do not trust the government to do the right thing” (1 = strongly disagree to 5 = strongly agree) (the US, *M* = 3.39, *SD* = 1.22; Singapore, *M* = 2.48, *SD* = 1.05). A higher score suggests greater distrust.

Lastly, we measured respondents' cognitive ability items using the alternate cognitive reflection test (Toplak et al., 2014). The participants were asked four questions - sample question include, “If John can drink one barrel of water in 6 days, and Mary can drink one barrel of water in 12 days, how long would it take them to drink one barrel of water together? _____ days [correct answer: 4 days; intuitive answer: 9]. The correct answers were summed to create an index of cognitive ability (the US, *Median* = 1, *SD* = 1.02; Singapore, *Median* = 1, *SD* = 1.32).

## 3 Results

At the first step, we compared statistical differences between control and treatment groups in terms of age, gender, education, income, and cognitive ability. The results from both countries suggest no characteristic differences between the two groups.

Once confirmed that there were no inherent differences between the groups, we ran independent *t*-tests to test the hypothesis (*H1*). The results suggested that in the US, participants exposed to the deepfake stimulus demonstrated (*M* = 3.48, *SD* = 1.26) significantly higher mistrust in the government than participants in the control group (*M* = 3.19, *SD* = 1.12), *t*_(301)_ = 2.02, *p* = 0.04. The finding was associated with a effect size, *d* = 0.24, indicating that exposure to the deepfake stimulus was linked to a modest increase in distrust in government (see Supplementary material B for details). No such differences were found in Singapore (deepfake condition: *M* = 2.46, *SD* = 1.03, control group: *M* = 2.51, *SD* = 1.10, *t*_(308)_ = −0.37, *p* = 0.71). The results confirm the potential adverse effects of deepfakes on political trust in the US but not Singapore.

**Figure 2:**
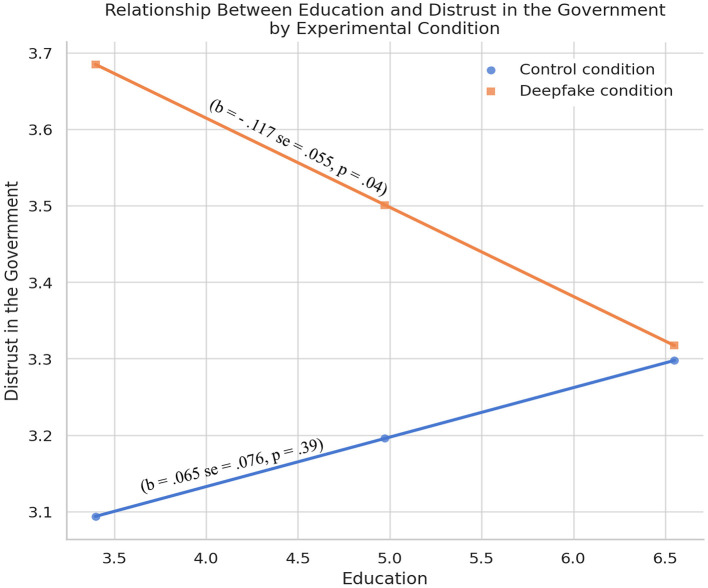
The moderation between education and conditions (control vs. deepfake) in predicting political distrust in the US.

We ran statistical tests to examine the moderating effect of cognitive ability in both countries to test if the impact of deepfake on political trust is contingent upon users' cognitive ability. However, no significant effects were observed across both countries. To proxy cognitive ability, we tested whether educational attainment moderates the impact of experimental conditions on governmental distrust. The results are included in Table 1. The results suggest that the interaction between education and conditions in predicting political distrust is statistically significant (*B* = −0.181, *SE* = 0.093, *p* = 0.05). The relationship is plotted in Figure 2.

**Table 1:** Predicting political distrust in the US (moderation between conditions and education).

**Predictor**	***b***	***Se***	***p***
Education	0.246	0.160	0.13
Condition (*ref* = control condition)	1.208	0.478	0.01
Condition x Education	−0.181	0.093	0.05
Age	0.009	0.004	0.04
Gender	0.281	0.152	0.07
Income	0.011	0.018	0.54
*R* ^2^	0.062^*^		

^*^p < 0.05.

In the control condition, the association is weak and not statistically significant (*b* = 0.065, *SE* = 0.076, *p* = 0.39), suggesting education has little effect on distrust when no deepfake is shown. In the deepfake condition, the relationship is significant and negative (*b* = −0.117, *SE* = 0.055, *p* = 0.04), indicating that more educated individuals expressed less distrust after exposure. This suggests education may act as a buffer against the persuasive impact of synthetic media. Together, these results point to a potential moderating role of education in shaping how people respond to deepfakes in the US.

None of the other demographic factors (gender, education, and income) were found to be statistically significant moderators.

## 4 Discussion

This study examined how deepfakes impact citizens' trust in their government across two distinct cultural and political contexts: the United States and Singapore. In contrast to Singapore, which represents a stable Asian political system, the U.S. reflects a Western political system characterized by significant polarization (e.g., Bellows, 2009; McCarty et al., 2016). These contrasts provide a unique cross-national framework to test the effects of deepfakes on political trust.

The findings indicate that exposure to deepfakes depicting infrastructural failures is associated with a lower trust in the government in the U.S., but this relationship is not significant in Singapore. This suggests that deepfakes are more destabilizing for uninformed citizens in the U.S. than in Singapore. Trust in government is essential for societal functioning, especially during crises and uncertainty. For example, during the COVID-19 pandemic, societies marked by polarization and low trust in government responded less effectively to the crisis (Liu et al., 2021; Vardavas et al., 2021). In the U.S. context, the findings suggest that citizens are particularly vulnerable to deepfakes, aligning with previous research (Chesney and Citron, 2018; Köbis et al., 2021). Growing disinformation has further eroded trust in the U.S. government (Citrin and Stoker, 2018), including deepfakes, which can potentially reduce public support for government actions, as observed in this study. This pattern also reflects contextual factors in the U.S., such as heightened political polarization, fragmented media environments, and widespread skepticism toward government institutions (Flynn et al., 2017; Lazer et al., 2018). These conditions may increase receptivity to disinformation, including deepfakes. While we do not make causal claims about cross-national differences, the findings suggest that institutional context, particularly levels of trust, may shape how citizens respond to deceptive media.

In contrast, the lack of significant adverse effects in Singapore can be explained by several factors. First, public trust in the Singaporean government is relatively high compared to many other countries (Min, 2023), making it less susceptible to erosion by deepfakes. In our sample itself, among the control condition, those in Singapore exhibited lower distrust than those in the US (see Supplementary material C for details). Second, Singapore's small geographical size means citizens are more familiar with their surroundings. This familiarity may have enabled some participants to recognize that the bridge collapse depicted in the stimulus was fabricated, limiting the impact of the deepfake. Third, Singapore enjoys a high rank in infrastructure development where failures are a rarity. Therefore, it would perhaps be relatively easier for the participants to reconsider the fabrication.

Next, our findings show that the moderation effect of cognitive ability is insignificant, suggesting that protective factors potentially curbing deepfake susceptibility do not matter in this case. However, education was found to be a significant moderator. These findings imply that educational attainment serves as a specific buffer against the destructive effects of deepfakes on political trust rather than driving a general predisposition toward skepticism. In the absence of manipulated media (the control condition), education has no discernible impact on distrust, but under deepfake exposure, more educated individuals register significantly less distrust. Practically, it underscores the importance of embedding digital and media-literacy training within formal education and public outreach programs to bolster collective resilience to AI-generated misinformation.

As governments and educational institutions acknowledge the harmful impact of deepfakes highlighted in this study, promoting media literacy will be a critical strategy for building societal resilience against the challenges posed by deepfakes, particularly those generated by generative AI (GenAI). As media literacy education gains prominence in academic curricula and public discourse, it will strengthen citizens' cognitive preparedness, enhancing their ability to critically assess and resist deepfakes and disinformation (Mihailidis and Viotty, 2017). More specifically, tailored interventions—such as interactive fact-checking tools, simulation-based workshops, and easy-to-access verification resources—could help lower-education audiences develop similar safeguards.

In sum, this study investigated how novel deepfakes undermine public trust in the government. It showed that deepfakes significantly reduce civic trust in the government in the US but not in Singapore. Despite its experimental design and novel approach, one key limitation needs to be considered. Since there were slight variations in the stimulus used across the two countries, with an emphasis on curating each image to the context, direct comparison should be taken with caution. However, future research can explore a single deepfake that is relatable to more than one country at a time. Additionally, this study did not include a formal stimulus validation, limiting our ability to confirm participants' perceptions of realism. Similarly, the absence of a neutral control condition restricts baseline comparison. We acknowledge these as methodological limitations and recommend their inclusion in future research. Finally, trust in government was measured using a single item, which limits construct validity. Future research should employ multi-item scales to better capture the multidimensional nature of political trust.

## Data Availability

The raw data supporting the conclusions of this article will be made available by the authors, without undue reservation.
