# Supplementary material for: False failures, real distrust: the impact of an infrastructure failure deepfake on government trust
Source: Front Psychol. 2025 May 23;16:1574840. doi: 10.3389/fpsyg.2025.1574840 (PMC12141277; doi:10.3389/fpsyg.2025.1574840)

Supplementary A: Sample Power Analysis

Sensitivity analysis (G*Power 3.1.9.7; Faul et al., 2007) indicated that given N = 303 (df = 301) in the US and N = 310 (df = 308) in Singapore, a two-tailed α=.05 and power = 0.95, the minimum detectable effect size was d = 0.439 in the US and d = 0.441 in Singapore and (t = 1.97). Thus, our design was well-powered to detect medium or larger effects (d≥.44) but would have had less than 95% power to detect smaller effects in both countries.

Supplementary B: Effect Size

The finding was associated with an effect size, *d* = 0.24, indicating that exposure to the deepfake stimulus was linked to a modest increase in distrust in government. While this effect size is considered small by traditional benchmarks (Cohen, 1988), more recent scholarship suggests that such magnitudes are typical and meaningful within this area of research (e.g., Pennycook & Rand, 2019). For example, Gignac and Szodorai (2016) note that in individual differences research, correlations of .10, .20, and .30 can be interpreted as small, medium, and large, respectively—placing our observed effect within the expected range. Given the subtle nature of the manipulation and the growing prevalence of synthetic media, even small effects may carry important theoretical and practical implications. As such, the observed effect size aligns with patterns commonly reported in this literature and should not be dismissed as small.

Supplementary C: Political Trust in Singapore v the US

An independent samples *t*-test was conducted to compare political distrust between control condition participants in Singapore and the US. There was a significant difference in scores between the two groups (t = -4.40, p = .000, d = 0.62). Those in Singapore (M = 2.51, SD = 1.10) exhibited lower distrust than those in the US (M = 3.20, SD = 1.12).

Supplementary D: Box Plot across Country and Conditions for Political Distrust

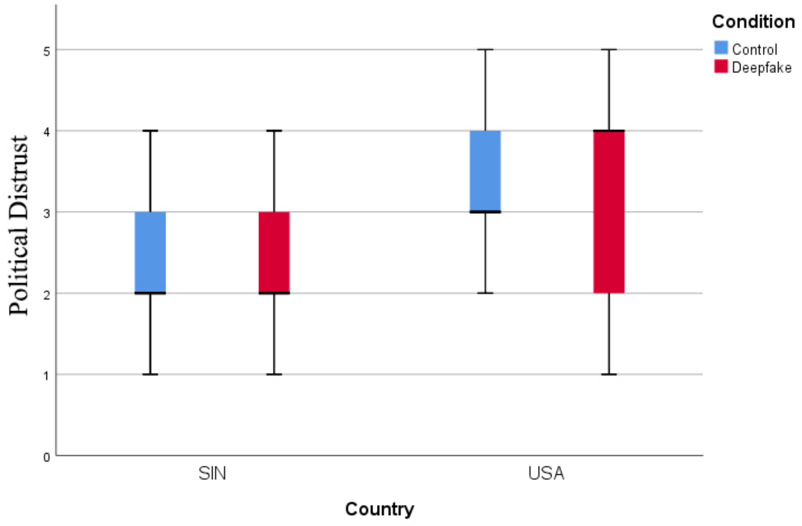

Supplement: Supplementary file 1 [file Data_Sheet_1.pdf]
